# Particulate matter and atherosclerosis: role of particle size, composition and oxidative stress

**DOI:** 10.1186/1743-8977-6-24

**Published:** 2009-09-18

**Authors:** Jesus A Araujo, Andre E Nel

**Affiliations:** 1 Division of Cardiology, Department of Medicine, David Geffen School of Medicine, University of California, Los Angeles, CA 90095, USA; 2 Division of Nanomedicine, Department of Medicine, David Geffen School of Medicine, University of California, Los Angeles, CA 90095, USA

## Abstract

Air Pollution has been associated with significant adverse health effects leading to increased morbidity and mortality. Cumulative epidemiological and experimental data have shown that exposure to air pollutants lead to increased cardiovascular ischemic events and enhanced atherosclerosis. It appears that these associations are much stronger with the air particulate matter (PM) component and that in urban areas, the smaller particles could be more pathogenic, as a result of their greater propensity to induce systemic prooxidant and proinflammatory effects. Much is still unknown about the toxicology of ambient particulates as well as the pathogenic mechanisms responsible for the induction of adverse cardiovascular health effects. It is expected that better understanding of these effects will have large implications and may lead to the formulation and implementation of new regulatory policies. Indeed, we have found that ultrafine particles (<0.18 μm) enhance early atherosclerosis, partly due to their high content in redox cycling chemicals and their ability to synergize with known proatherogenic mediators in the promotion of tissue oxidative stress. These changes take place in parallel with increased evidence of phase 2 enzymes expression, via the electrophile-sensitive transcription factor, p45-NFE2 related transcription factor 2 (Nrf2). Exposure to ultrafine particles also results in alterations of the plasma HDL anti-inflammatory function that could be indicative of systemic proatherogenic effects. This article reviews the epidemiological, clinical and experimental animal evidence that support the association of particulate matter with atherogenesis. It also discusses the possible pathogenic mechanisms involved, the physicochemical variables that may be of importance in the greater toxicity exhibited by a small particle size, interaction with genes and other proatherogenic factors as well as important elements to consider in the design of future mechanistic studies.

Extensive epidemiological evidence supports the association of air pollution with adverse health effects [1-3]. It is increasingly being recognized that such effects lead to enhanced morbidity and mortality, mostly due to exacerbation of cardiovascular diseases and predominantly those of ischemic character [4]. Indeed, in addition to the classical risk factors such as serum lipids, smoking, hypertension, aging, gender, family history, physical inactivity and diet, recent data have implicated air pollution as an important additional risk factor for atherosclerosis. This has been the subject of extensive reviews [5,6] and a consensus statement from the American Heart Association [7]. This article reviews the supporting epidemiological and animal data, possible pathogenic mechanisms and future perspectives.

## Exposure to air particulate matter leads to increased cardiovascular morbidity and mortality

While air pollution is a complex mixture of compounds in gaseous (ozone, CO and nitrogen oxides) and particle phases [8], the strongest evidence among several hundred epidemiological studies linking air pollution with human health effects, centers around the particulate components [7-13]. Particulate matter (PM) is comprised of heterogenous compounds varying in size, number, chemical composition, surface area, concentration and source [7,8]. Some atmospheric particles are liquid, some are solid and others may contain a solid core surrounded by liquid. PM includes primary particles that are emitted directly from sources such as fossil-fuel combustion (e.g. diesel exhaust particles) and secondary particles that are generated from gases through chemical reactions involving atmospheric oxygen (O_2_), water vapor (H_2_O), reactive species such as ozone (O_3_), free radicals such as hydroxyl (.OH) and nitrate (.NO3) radicals, pollutants such as sulfur dioxide (SO_2_), nitrogen oxides (NOx), and organic gases from natural and anthropogenic sources [8].

**Figure 1:**
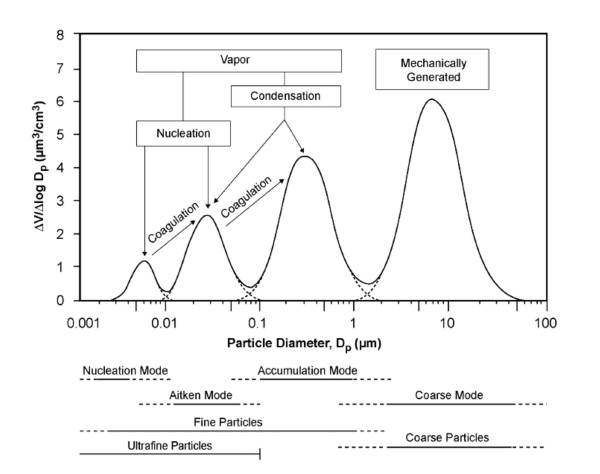
**Idealized particle size distribution that might be observed in traffic**. Particles from different sizes are generated by four modes: nucleation, aitken, accumulation and coarse mode. Also shown are the major formation and growth mechanisms of the four modes of ambient particles. V = volume, D_p _= particle diameter. Source: U.S. EPA [8].

Particles are also classified according to their aerodynamic diameter into size fractions such as PM_10 _("thoracic" particles, < 10 μm), PM_2.5-10 _("coarse" particles, 2.5 to 10 μm), PM_2.5 _(fine particles, < 2.5 μm and UFP (ultrafine particles, < 0.1 μm). These particles are derived from various sources and by various mechanisms as shown in Table 1, producing distinct lognormal modes in the particle size distributions by number and volume (nucleation, Aitken mode, accumulation and coarse modes) (Figure 1) [8]. UFP correspond to the nucleation and Aitken modes. They are typically derived from combustion of fossil-fuels and are the result of nucleation of gas phase species to form condensed phase species in newly formed particles which have had little chance to grow (nucleation mode) or in recently formed particles that are actively undergoing coagulation (Aitken mode). Some particle fractions are inclusive of others like PM_2.5 _which contain particles in the nucleation and Aitken mode (UFP) as well as particles in the accumulation mode (> 0.1 μm), where particles grow in size and "accumulate" by coagulation (two particles combining to form one) or by condensation (gas molecules condensing on a particle). The coarse particles correspond to the coarse mode, typically derived from soil, agricultural and road dust, tire wear emissions, construction debris, mining operations and are mainly formed by the mechanical breakdown (crushing, grinding, abrasion of surfaces) of crustal material, minerals and organic debris [8]. Major sources of PM_2.5 _include power plants, oil refinery and metal processing facilities, wildfires, tailpipe and brake emissions from mobile sources, residential fuel combusion. UFP are mostly generated through tailpipe emissions from mobile sources (motor vehicles, aircrafts, and marine vessels) [14].

**Table 1:** Classification of particles based on size

**Particle**	**Aerodynamic diameter (μm)**	**Sources**	**Mode of generation**	**Atmospheric half-life**
Thoracic particles(PM_10_)	< 10	-	-	-

Coarse particles(PM_2.5-10_)	2.5 - 10	Suspension from disturbed soil (farming, mining, unpaved roads), construction, plant and animal fragments	Mechanical disruption (crushing, grinding, abrasion of surfaces), evaporation of sprays, suspension of dusts.	Minutes to hours

Fine particles (PM_2.5_)	< 2.5	Power plants, oil refineries, wildfires, residential fuel combustion, tailpipe and brake emissions	Gas-to-particle conversion by condensation, coagulation (accumulation mode)	Days to weeks

Ultrafine particles (UFP)	< 0.1*	Fuel combustion (diesel, gasoline) and tailpipe emissions from mobile sources (motor vehicles, aircrafts, ships)	Fresh emissions, secondary photochemical reactions (nucleation mode)	Minutes to hours

PM_10 _includes all other PM fractions. * While the UFP size cutoff is by definition considered < 0.1 μm, UFP can include particles up to 0.2 μm in experimental studies using particle concentrators, because the distinction between the ultrafine and the accumulation modes can vary from 0.1 to 0.2 μm depending upon location and season for reasons that include among others, variability in the size of particulate derived from "fresh" emission sources and deviation from a spherical shape [101]. Table based on U.S. EPA [8].

Multiple studies show that short-term exposure to PM_10 _results in small increases in cardiovascular mortality. Thus, in approximately 50 million people from NMMAPS (National Morbidity, Mortality, and Air Pollution Study), it was found that daily cardiopulmonary mortality increased by 0.6% for every 20 μg/m^3 ^increase in PM_10 _load [15]. This association was confirmed in Europe, where ~43 million adults in 29 European cities from the APHEA2 (Air Pollution and Health: A European Approach) study exhibited a 1.5% increase in daily cardiovascular mortality for every 20 μg/m^3 ^increase in PM_10 _[16]. However, studies have also shown that the magnitude of these associations are bigger with the smaller PM_2.5 _fraction. Thus, 12,865 patients enrolled in IHCS (Intermountain Heart Collaborative Study) had a daily increase of 4.5% in acute ischemic coronary events per 10 μg/m^3 ^increase in ambient PM_2.5 _[17]. Associations of air pollution with cardiovascular events have also been demonstrated in longer-term exposure studies. The latest report of the American Cancer Society II study demonstrated in ~500,000 adults, a 12% increase in cardiovacular deaths per 10 μg/m^3 ^increase in long-term (annual average) PM_2.5 _exposure [4]. Importantly, this study showed that deaths from ischemic heart disease was the single largest cause of mortality (18% increase). This association was even stronger in the extended analysis of the Harvard Six Cities Study that showed a 28% increase in cardiovascular (CV) deaths per 10 μg/m^3 ^increase in long-term PM_2.5 _exposure [18], and even stronger in the Women Health Initiative Study [19], which showed in 65,893 post-menopasual women, a 24% and 76% increase in the incidence of CV events and CV mortality, respectively. It appears that as the studies have gotten better tailored to assess CV morbility and mortality, the strength and magnitude of associations have become stronger. In addition, the extended analysis of the Harvard Six Cities Study showed in 8,096 participants from six US cities followed up over two time periods (1974 through 1989 and 1990-1998) that a decrease in CV mortality paralleled a decline in annual mean PM_2.5 _concentrations. CV mortality exhibited a 31% reduction per each 10-μg/m^3 ^improvement in city-specific mean PM_2.5 _in between the two periods [18]. More importantly, the drop in the adjusted mortality rate was largest in the cities with the largest reductions in PM_2.5_, underscoring the importance of PM monitoring, regulation and acting upon this problem, given the potential and substantial benefits in Public Health outcomes. It has been suggested that reduction in the level of air PM may even account for some of the reduction observed in US mortality rates over the last 2 decades [20]. Although the epidemiological studies with PM_10 _and PM_2.5 _data support the notion that a smaller particle size correlates with bigger cardiovascular effects, there are few reports supporting the association of UFP with increased total or cardio-respiratory mortality [21,22]. Lack of studies is partly due to the difficulty of quantifying UFP exposure due to the lack of routine air pollution monitoring equipment to assess UFP parameters.

**Figure 2:**
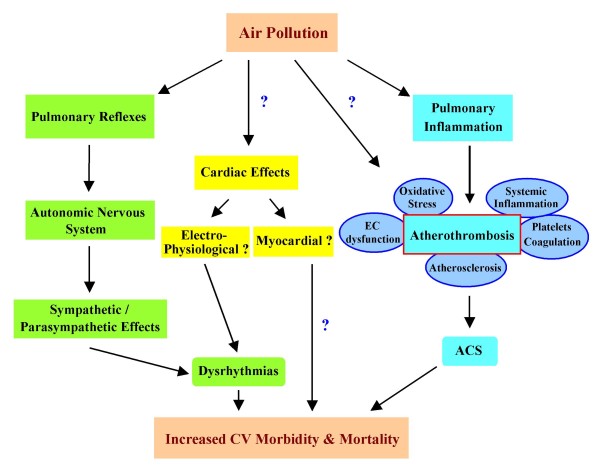
**Possible mechanisms that link air pollution with increased cardiovascular morbidity and mortality**. Air pollutants may trigger pulmonary reflexes that can activate the autonomic nervous system and result in alterations of heart rate variability and induction of dysrhythmias (in green). Air pollutants may directly affect cardiac tissue leading to the induction of dysrhythmias and/or cardiomyopathy (in yellow). Air pollutants can also enhance atherothrombotic processes that lead to the development of acute coronary syndromes (ACS) via the generation of pulmonary inflammation or direct translocation into the systemic circulation (in blue). Atherothrombotic processes include increased vascular oxidative stress, endothelial cell dysfunction, atherosclerosis and increased platelet aggregability and coagulation, among others.

Exposure to ambient PM leads to enhanced CV morbidity and mortality by a variety of proposed mechanisms, summarized in Figure 2. CV ischemic events may be due to a myriad of both acute and chronic effects. Thus, acute exposure to PM has been associated with the triggering of acute myocardial infarction [23-25], discharge of implanted automatic cardioverter defibrillators [26], hospitalizations for acutely decompensated congestive heart failure [27] and ischemic stroke [28]. Such acute effects may involve prothrombotic mechanisms such as elevation of fibrinogen [29], increased platelet aggregation and factors II, VII and X activity [30] or alterations of arterial vasoconstriction [13], leading to greater propensity for the development of ischemia in subjects with coronary artery disease [31,32]. The accumulation of acute and sub-acute effects or the chronic exposure to PM may also result in the promotion of atherosclerosis which can be evidenced over months or years.

The first study to find an association of PM exposure with atherosclerosis in human subjects was performed in the University of Southern California by Kunzli et al [33], a cross-sectional study that included 798 individuals. They correlated the degree of carotid intima-media thickness (CIMT) with levels of annual mean concentrations of ambient PM_2.5_, estimated by geocoding of the subjects' residential areas (Table 2). This study showed a 5.9% increase in carotid intima-medial thickness for every 10 μg/m^3 ^rise in PM_2.5 _levels [33]. Likewise, a prospective German cohort study supported an association in between long-term residential exposure to high traffic, levels of PM_2.5 _and coronary atherosclerosis as assessed by coronary artery calcification scores [34]. Hoffmann et al found in 4,494 participants, that compared with subjects living > 200 meters away to a major road, subjects living within 50, 51 to 100 and 101 to 200 meters showed 63%, 34% and 8% increase in the probability of having a high coronary artery calcification (CAC) score, respectively. Nevertheless, the strength of associations between PM and atherosclerosis in humans is still being debated. While Hoffmann et al found positive associations with the distance to a major road, there were non-statistically significant associations with PM_2.5 _exposure. In addition, data from the Multi-Ethnic Study of Atherosclerosis [35] show some weaker associations than those shown by the previous studies (Table 2). Hence, long-term (20-year) exposure to PM was estimated on the basis of a residential history and the use of a spatio-temporal model to predict PM_10 _and PM_2.5 _exposures for each participant-month based on the geographic location of each participant's residence each month. Three measures of subclinical atherosclerosis, such as CIMT, coronary artery calcification and ankle-brachial index (ABI) were employed in 5,172 US participants. While no associations were present with either coronary calcium or ABI measures, PM_10 _exposures (20-year means and 2001 mean) and 20-year PM_2.5 _exposures did correlate with 1-3% increase in CIMT per 21 μg/m^3 ^increase in PM_10 _or 12.5 μg/m^3 ^increase in PM_2.5_, respectively.

**Table 2:** Human studies linking air pollution exposure with atherosclerosis

**Study**	**Air pollutant**	**Evaluation of atherosclerosis**	**Major Findings**	**Reference**
Kunzli et al	PM_2.5_ Ozone	CIMT	5.9% increased in CIMT per every 10 μg PM_2.5_/m^3^	[33]

Hoffmann et al	PM_2.5_ Distance to major road	CACS	Increased CAC scores with shorter distances to a major road	[34]

Diez Roux et al	PM_10_ PM_2.5_	CIMT CACS BAI	1-3% increase in CIMT per every 21 and 12.5 μg/m^3 ^in PM_10 _and PM_2.5 _respectively	[35]

CIMT = Carotid intima-media thickness, CACS = coronary artery calcium score,

BAI = Brachial artery index.

Based on this evidence, it would appear that PM_2.5 _exposure may contribute to enhancement of human atherosclerosis, although the strength and relative importance of this mechanism in contributing to cardiovascular morbidity and mortality is still to be ascertained. Just as the strength of the associations of PM with cardiovascular outcomes increased when moving from PM_10 _to PM_2.5 _metrics, it has been suggested that these associations could be even stronger with the UFP fractions for reasons that are discussed below. Although there are no studies available yet that have evaluated the effects of UFP exposures on human atherosclerosis, recent findings from the Southern California Particle Center (SCPC) are consistent with the notion of UFP's greater proatherogenic potential. Delfino et al studied residents in independent living facilities of two retirement communities in Los Angeles [36]. 31 subjects with history of CAD were followed up over 7-month periods with a very detailed pollutant exposure characterization and blood collection for the determination of systemic inflammatory, antioxidant and coagulation markers. They found positive associations of particle number (dominated by UFPs) and outdoor quasi-ultrafine PM_0.25 _(< 0.25 μm) with biomarkers of systemic inflammation such as C-reactive protein (CRP), interleukin (IL-6) and soluble tumour necrosis factor receptor II (sTNF-RII). By contrast, 4-day average outdoor accumulation-mode PM_0.25-2.5 _showed an unexpected inverse association instead [36]. This study is in agreement with a previous report where exposure to UFP correlated with increased plasma levels of soluble CD40 ligand (sCD40L), a marker for platelet activation that reflects increased coagulation and inflammation [37]. Thus, there is need for human studies that evaluate the potential links between UFP exposures and clinical measures of atherosclerosis.

## Studies in animal models support a causal association for PM and atherosclerosis

Work with experimental animals have been important to establish a causal association between exposure to PM and atherosclerosis. The first evidence was from work at the University of British Columbia in Ontario, Canada [38]. Suwa et al exposed female Watanabe heritable hyperlipidemic rabbits to biweekly intrapharingeal instillations of urban air PM_10_. The degree of aortic and coronary atherosclerosis was evaluated by estimating the atherosclerotic lesion volumes in sections from the various vessels. Qualitative analysis included the assessment of the types of plaques according to AHA guidelines [39,40], plaque composition and cellularity. They determined that although there were no differences in the frequency of plaques in either the coronaries or aorta, PM_10 _administration resulted in a 71% significant increase in the relative volume of atherosclerotic lesions in the coronary arteries and a 62% increase in the relative volume of extracellular lipids in the aorta. Plaques were also more advanced and with a greater content of smooth muscle cells in the coronaries. Interestingly, the burden of atherosclerotic lesions correlated with the percentage of alveolar macrophages positive for particles.

**Figure 3:**
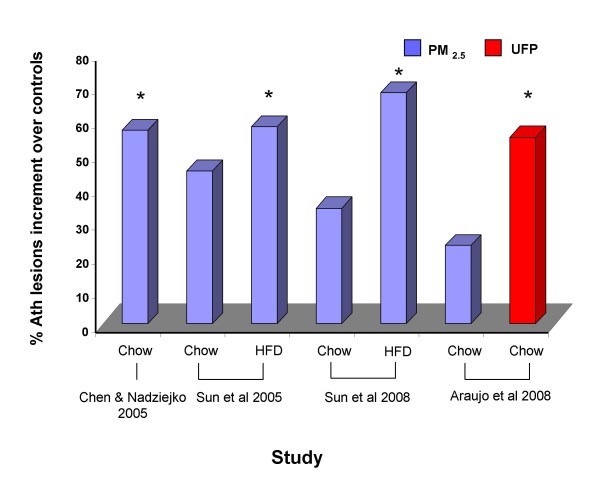
**PM effects on atherosclerotic lesions in animal studies**. The relative increment in atherosclerotic lesions induced by PM exposures over filtered-air controls is displayed (%) Atherosclerotic lesions were assessed by histological analysis of aortic cross-sections, en-face assessment of the whole aorta or ultrasound biomicroscopy depending on the study. Mode of atherosclerotic lesion assessment and references for each study are shown in Table 3. HFD = High fat diet. * p < 0.05 in comparison with the corresponding filtered-air controls.

The effects of inhaled PM_2.5 _on aortic atherosclerosis have been assessed on apoE null mice subject to long-term exposures to concentrated ambient particles (CAPs) performed at the A.J. Lanza Exposure Laboratory of New York University in Tuxedo, NY (Table 3). The first study reported in 2005 showed that 39-41 week-old apoE^-/- ^mice fed a chow diet and exposed to 10× ambient concentrations of PM_2.5 _for 6 hours per day, 5 days per week for 5 months exhibited a 57% increase in the percentage of atherosclerotic plaque area in the aortic root [41] (Table 3, Figure 3). However, there were no differential effects in the context of extreme hyperlipidemia exhibited by apoE^-/-^, LDL^-/- ^mice exposed to the same conditions [41]. A second study reported the same year showed that younger 6-week-old apoE^-/- ^mice fed a chow diet and exposed to similar conditions for 6 months displayed a 45% increase in the percentage of aortic atherosclerotic plaque area that was not statistically significant [42]. In this study, however, marked hyperlipidemia achieved by the feeding of a high fat diet resulted in a greater and significant promotion of atherosclerosis as CAPs-exposed mice showed a 58% significant increase in aortic root plaque area (Figure 3) [42], together with impaired vasomotor response. More recently, a third study of apoE^-/- ^mice fed a high fat diet and exposed to PM_2.5 _in the same facility confirmed the enhancement of aortic atherosclerosis as assessed by the percentage of the plaque area in the aortic arch by ultrasound bio-microscopy [43] (Table 3, Figure 3). In this study, PM_2.5 _exposures also led to atherosclerotic plaques that were richer in tissue factor [43], which could play a causative role or simply be an indicator of greater atherosclerotic plaque burden. One limitation of these studies is the relatively small number of sex-matched animals (n < 10/group) used to assess vascular pathology that has a large phenotypic variance. However, the consistent reproducibility of PM_2.5 _proatherogenic effects in all three studies decrease the potential for type I error.

**Table 3:** Animal studies evaluating the effect of air pollution on atherosclerosis

**Study**	**PM fraction (mode of administration)**	**Animal model**	**Diet**	**Assessment of Atherosclerosis (Method)**	**Effect on atherosclerosis**	**Reference**
Suwa et al 2002	PM_10 _(I.T.)	Watanabe rabbits	Chow	% lesional volume in coronary arteries and aorta (histology)	Increase	[38]

Chen & Nadziejko 2005	PM_2.5_ (Inhaled CAPs)	apoE^-/-^, LDL^-/- ^mice apoE^-/- ^mice	Chow Chow	% lesional area in whole aorta (histology)	No change Increase	[41]

Sun et al 2005	PM_2.5_ (Inhaled CAPs)	apoE^-/- ^mice	Chow HFD	% lesional area in cross-sections of aorta (histology)	N.S. increase Increase	[42]

Sun et al 2008	PM_2.5_ (Inhaled CAPs)	apoE^-/- ^mice	Chow HFD	% lesional area in aorta (ultrasound)	N.S. increase Increase	[43]

Araujo et al 2008	PM_2.5 _& UFP (Inhaled CAPs)	apoE^-/-^mice	Chow Chow	Mean lesional area in aortic root (histology)	N.S. increase Increase	[44]

I.T. = Intratraqueal, CAPs = Concentrated ambient particles, HFD = High fat diet, N.S. = Not significant

In another animal study in the Southern California Particle Center space (SCPC) and the University of California in Los Angeles (Table 3) [44], we tested the notion that the oxidant potential and size of ambient particles play an important role in determining atherosclerotic lesion development, with a hypothesis that UFP should trigger more plaque development than bigger particles thanks to their greater prooxidant potential, as it will be discussed below. Araujo et al exposed apoE^-/- ^mice fed a chow diet to concentrated PM_2.5_, UFP or filtered air for 5 hours/day, 3 days per week for 5 weeks. UFP-exposed mice developed 25% and 55% more aortic atherosclerosis, assessed by the mean atherosclerotic lesional area in the aortic root, compared to PM_2.5 _or FA-exposed mice, respectively [44]. Although PM_2.5 _exposures tended to enhance lesion formation over FA controls, resulting in a 23% increment in lesional area, these effects were relatively smaller than in the previous studies (Figure 3). One possible explanation could be the shorter duration of exposures (5 weeks instead of 5-6 months). This is exemplified by the data in Figure 3 showing that the degree of PM_2.5_-induced atherogenesis increases with the length of the exposures. In addition, the magnitude of these effects is enhanced by UFP exposures to yield a comparable degree of atherosclerosis enhancement as long-term PM_2.5 _exposures (Figure 3).

One limitation of the SCPC study is that the concentrator technology employed (VACES: Versatile Aerosol Concentration Enrichment System) generates overlapping CAPs aerosols. The fact that the sub-2.5 μm aerosol included UFP made straight comparison with the UFP aerosol difficult since particle mass in the former aerosol was mostly determined by the accumulation mode particles (0.1-2.5 μm). The more abundant UFP contributed to less than a fifth of the PM_2.5 _aerosol's total mass. It is possible that the true relative proatherogenic effects of UFP, compared to the larger 0.1-2.5 μm particles are bigger than can be inferred from our study. When normalized by mass, atherosclerotic lesion scores of UFP-exposed mice were 460% greater than scores exhibited by PM_2.5_-exposed mice, an extrapolation that is not entirely adequate given that both aerosols overlapped in size. On the other hand, the UFP aerosol contained 44% more particles than the number of sub-0.18 μm particles present in the PM_2.5 _aerosol which could explain the observed difference, provided that the sub-0.18 μm components accounted for all or most of the PM_2.5_-induced proatherogenic effects. Any of these extrapolations could enhance the approximately 25% difference in the proatherogenic potentials of UFP versus the larger 0.1-2.5 μm particles. An accurate estimate of the relative potency of UFP vs. 0.1-2.5 μm particles would require a different concentrator technology with selective exposures to UFP and 0.1-2.5 μm particles as well as the use of four parallel exposure groups where both particle mass and particle number concentrations were matched to quantify the relative importance of UFP.

An additional limitation of this study is that it only evaluated a single dose at only one time point, which impedes the assessment of threshold, latency of effects or potency. Furthermore, the particles collected in Los Angeles have not been compared to other locations in the world where unique aerosol compositions in each size range could have different outcomes. It will be important to conduct similar studies in different places where the effects of different particle compositions on atherosclerosis, not only size, can be assessed.

## Proposed oxidative stress paradigm for understanding of PM proatherogenic effects

A number of mechanisms have been proposed to explain the cardiovascular effects of ambient PM (Figure 2), which has been the subject of recent extensive reviews [5,6,45]. At the cellular level, these various mechanisms involve free radical production (by transition metals and organic compounds), oxidative stress, cytokine release, inflammation, endotoxin-mediated damage, stimulation of capsaicin receptors, autonomic nervous system activity, covalent modification of key cellular molecules and increased pro-coagulant activity [5,6,45,46].

**Figure 4:**
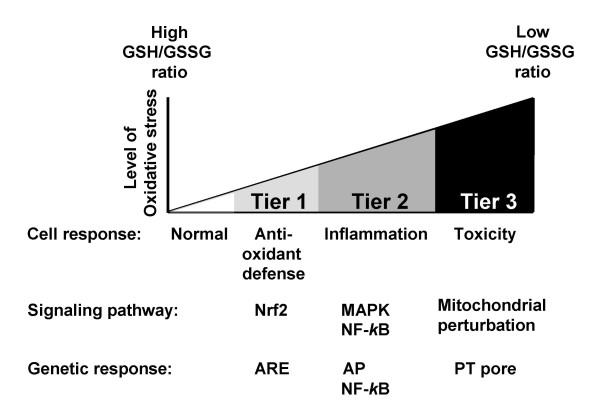
**Stratified hierarchical response to air pollutants**. Macrophages exposed to particulate matter components (e.g. DEP) respond in a dose-dependent fashion via the activation of pathways and mechanisms at various levels of action (tiers). For example, Nrf2 can induce the transcription of antioxidant genes via the antioxidant response element (ARE) in the earliest level of defense (tier 1). When the mechanisms of defense involved in one tier are not sufficient to contain the injurious stimuli, signaling pathways of the subsequent(s) tier of action may get activated, such as MAPK pathways that regulate proinflammatory genes via Activating Protein (AP) transcription factors (tier 2) or proapoptotic signals triggered by mitochondrial perturbation that can lead to activation of the mitochondrial permeability transition (PT) pore and cellular toxicity (tier 3). Modified from Li et al [123].

Diesel exhaust particles (DEP) have been used as a model pollutant and extensively studied in airway inflammation. We and others have shown that the pro-inflammatory effects of DEP and concentrated air particles (CAPs), that result in enhancement of allergic inflammation [47,48], acute asthma exacerbation and bronchitis flares [3,49] are linked to ROS production [46,48,50]. This includes O_2_.^- ^production in macrophages, bronchial epithelial cells and lung microsomes incubated with the particles or their organic extracts [51,52]. Moreover, we have demonstrated that thiol antioxidants, which are highly effective in suppressing reactive oxygen species (ROS) production by organic DEP chemicals *in vitro*, interfere in the adjuvant effects of DEP in a murine allergen sensitization model [53]. We have shown evidence that DEP induce oxidative stress and cellular effects in target cells that can be better understood according to a stratified oxidative stress model, aka as the hierarchical oxidative stress hypothesis [54] (Figure 4). When ROS production overwhelms the antioxidant defenses, a state of cellular oxidative stress develops [55], characterized by a depletion of intracellular GSH, leading to GSSG accumulation and a drop in the GSH/GSSG ratio [55]. This state of redox disequilibrium initiates additional redox-sensitive signaling pathways, including activation of several MAP kinases as well as the NF-κB cascade. These cascades act in synergistic fashion to activate cytokine and adhesion molecule expression through appropriate promoter response elements. Indeed, a number of laboratories have demonstrated that DEP and ambient PM induce JNK, p38^MAPK ^and NF-κB activities, which are functionally linked to the cytokine and chemokine production *in vivo *and *in vitro *[56,57]. DEP has also been shown to elicit ROS production in vascular cells such as pulmonary artery endothelial cells [58], rat heart microvessel endothelial cells [59] and human microvascular endothelial cells [60]. Furthermore, it appears that DEP could induce oxidative stress, inflammatory and propapoptotic signals in endothelial cells in a dose-dependent hierarchical fashion as incremental DEP concentrations result in a larger recruitment of genes and toxicity [60].

**Figure 5:**
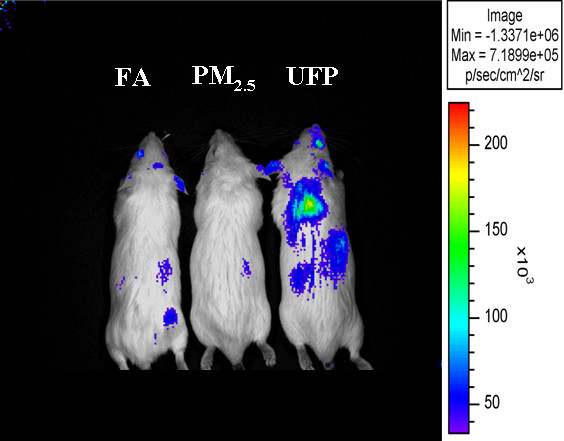
**Ambient UFP triggers prooxidant effects in-vivo**. Representative dorsal photograph of HO-*luc *transgenic mice that were exposed to concentrated UFP, concentrated PM_2.5 _or filtered-air (FA) for 5 hours in a mobile exposure laboratory located in downtown Los Angeles, ~300 meters away from the I-110 freeway. Increased bioluminescence was due to a larger HO-1 upregulation response generated in tissues subject to greater oxidative stress. Mice were generated and obtained from Dr. Christopher Contag at Stanford University [62]. They contain a modified coding sequence of the luciferase gene under the control of the full HO-1 promoter. Whole-body images were acquired within 3 hours of the exposure using a cooled charged-couple device (CCD) camera at the Small Animal Imaging Center of the UCLA Crump Institute for Molecular Imaging [124] as previously described [125]. The bioluminescence signal was recorded as maximum photons/sec/cm^2^/steradian (p/sec/cm^2^/sr). Notice that the UFP-exposed mouse displays increased luciferase emissions both in the thorax and abdomen as compared with the PM_2.5 _or FA-exposed mice.

We have shown that the aromatic and polar DEP fractions, which are enriched in polycyclic aromatic hydrocarbons (PAHs) and quinones, respectively, are most active in the induction of heme oxygenase 1 (HO-1), glutathione S-transferase (GST), and other phase II enzymes that correspond to the first tier of defense in macrophages (Figure 4) and epithelial cells [61]. We have also demonstrated that the triggering of this antioxidant defense is regulated by the transcription factor p45-NFE2 related transcription factor 2 (Nrf2) by modulation of its proteasomal degradation in the cytosol and translocation into the nucleus [61]. Interestingly, ambient PM also triggers Nrf2-regulated genes in response to its content of prooxidative and electrophilic chemicals. Various particle sizes have marked differences in their chemical composition as a result of their different sources of origin and modes of generation previously described. These differences result in different redox activity and ability to cause mitochondrial damage as shown in Table 4. Thus, ambient UFP triggers the induction of Nrf2-regulated HO-1 in macrophages to a greater degree than ambient PM_2.5 _or coarse particles [61]. The same can also be shown *in vivo *using a transgenic mouse model in which the heme oxygenase promoter was linked to a luciferase reporter gene [62]. Exposure of these animals in a mobile animal trailer showed that the promoter reporter gene construct is more readily induced in animals exposed to concentrated UFP compared to animals simultaneously exposed to a concentrated atmosphere of PM_2.5 _or filtered air (Figure 5). This greater responsiveness was in relation with the greater prooxidant potential of ultrafine PM due to their higher content of redox cycling organic compounds.

**Table 4:** Characteristics of PM fractions

**Parameter**	**Coarse (PM_2.5-10_)**	**Fine (PM_2.5_)**	**Ultrafine**
Size (μm)	2.5 - 10	0.15 - 2.5	< 0.15 μm

Number per μm^3^	+	++	+++

Mass (μg) per μm^3^	+++	++	+

Relative content (% of total mass) *			
Elemental carbon	+	++	+++

Organic carbon	+	++	+++

PAHs	+	+	+++

Metals	+++	++	+

Redox activity	+	++	+++

DTT assay **	+	++	+++

HO-1 induction^#^	+	++	+++

GSH depletion ^#^	+	+++	+++

Mitochondrial damage^#^	None	Some	Extensive

Surface area	+	++	+++

Bioavailability of active compounds	+	++	+++

Lung penetrability	+	++	+++

Modified from Li et al [123]. * Relative content was estimated using mass concentration and fractional composition of CAPs collected on Teflon and quartz filters in two locations from the Los Angeles basin as reported [67]. ** DTT assay was performed on similar CAPs samples [67]. # HO-1 induction, GSH depletion and mitochondrial damage were determined in a murine macrophage cell line (RAW 264.7) and a transformed human bronchial epithelial cell line (BEAS-2B) exposed to similar CAPs samples [67].

UFP exhibit greater relative content of elemental and organic carbon [44,63] that vary depending on the sources. Urban UFP contain a higher content per unit mass of polycyclic aromatic hydrocarbons (PAH), which are relevant organic constituents since they can induce oxidative stress and electrophilic chemistry in tissues [46,64] after conversion to quinones by the involvement of cytochrome P450 1A1, epoxide hydrolase and dihydrodiol dehydrogenase [65]. Some quinone species can act as redox cycling catalysts, leading to the formation of O_2_,^-^ when undergoing one-electron reductions by NADPH cytochrome P450 reductase to form semiquinones [66] and recycling to the original quinones [50,66]. There is an excellent correlation between the PAH content of UFP and their ability to engage in redox cycling reactions in-vitro. Indeed, UFP samples from the Los Angeles basin are more potent than PM_2.5 _or coarse PM at inducing oxidative stress as measured by the dithiothreitol (DTT) assay, extent of HO-1 induction, reduced glutathione to oxidized glutathione (GSH/GSSG) ratios and the ability to cause mitochondrial damage (Table 4). These biological endpoints are correlated to greater organic PM carbon and PAH contents, suggesting a role of these organic substances in generating redox activity [67-69]. Furthermore, UFP collected from various locations differ significantly in their ability to trigger Nrf2-regulated responses, also in strong relation with their chemical composition and electrophilic potential [67]. Thus, the greater prooxidant effects of the smaller particles appear to be partly derived from their higher content of prooxidant compounds.

**Figure 6:**
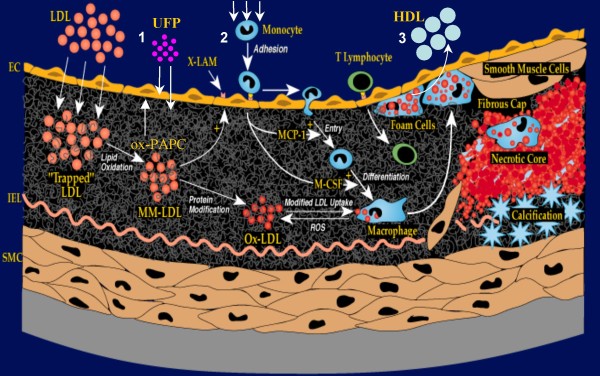
**Pathogenesis of atherosclerosis**. Lipid infiltration of the artery wall originating from circulating LDL followed by oxidative modification in the subendothelial space, monocyte chemotaxis and foam cell formation are among the earliest events in atherogenesis. Monocytes differentiate into macrophages, followed by release of inflammatory mediators and a vicious cycle of inflammation. More advance stages of the disease include smooth muscle cell proliferation, formation of fibrous caps, necrotic cores, calcification, rupture, hemorrhage and thrombosis. Possible mechanisms how PM enhances atherosclerosis include: 1) Systemically translocated UFP or their chemical constituents may synergize with ox-PAPC generated within ox-LDL in the activation of proatherogenic molecular pathways in endothelial cells, 2) Inflammatory mediators released from the lungs may promote monocyte chemotaxis into the vessels, 3) PM can induce HDL dysfunction with loss of its antiinflammatory properties. Modified from Araujo and Lusis [126].

PM ability to induce ROS generation appears to be key in promoting atherosclerosis, which could be the result of PM-mediated systemic prooxidant and proinflammatory effects. Atherosclerosis is a vascular inflammatory process where lipid deposition and oxidation in the artery wall constitutes a disease hallmark [39,40,70-73] (Figure 6). Infiltrating lipids come from low density lipoprotein (LDL) particles that travel into the arterial wall and get trapped in a three-dimensional cage-work of extracellular fibers and fibrils in the subendothelial space [74,75], where they are subject to oxidative modifications [76-78] leading to the generation of "minimally modified" LDL (mm-LDL). Such oxidized LDL is capable of activating the overlying endothelial cells to produce pro-inflammatory molecules such as adhesion molecules, macrophage colony-stimulating factor (M-CSF) and monocyte chemotactic protein-1 (MCP-1) [79-81] that contribute to atherogenesis by recruiting additional monocytes and inducing macrophage differentiation [70,71,73]. Later stages of the disease involve the proliferation of smooth muscle cells, formation of fibrous caps, induction of apoptosis, hemorrhage and calcification [73]. Animal models have been useful not only to demonstrate the PM proatherosclerotic effects as described in the previous section, but also to get some insights into the possible mechanisms involved. It is possible that the different proatherogenic effects of particles of different sizes and compositions could at least be partly related to their different abilities to elicit prooxidant and proinflammatory reactions in vascular cells.

## How do the prooxidative effects of PM promote atherogenesis?

The promotion of atherogenesis by PM is likely the result of systemic prooxidant and proinflammatory effects at vascular sites. In support of this notion, Nurkiewicz et al have shown that intratracheal instillation of residual oil fly ash (ROFA) [82,83] in rats led to greater vascular ROS generation, as assessed by the tetranitrobluetetrazolium (TNBT) reduction method [82], resulting in a dose-dependent impairment of systemic endothelium-dependent arterial dilation, increased leukocyte rolling and adhesion as well as deposition of myeloperoxidase in the spinotrapezius muscle microcirculation [82,83]. Likewise, long-term PM_2.5 _inhaled exposures that promote atherosclerosis, also lead to enhanced ROS generation in the aortic plaques and increased formation of 3-nitrotyrosine residues [42].

How does PM inhalation in the lung lead to the systemic effects on the vasculature?. One possibility is that prooxidant PM deposited in the lungs lead to the release of proinflammatory cytokines (Figure 6) into the blood stream by a series of steps such as: a) Deposition of inhaled particles in the respiratory tract, b) Ability to trigger or enhance free-radical production, c) Development of pulmonary inflammation, d) Release of inflammatory mediators into the systemic circulation. Inhaled small particles deposit in the respiratory tract via diffusion due to displacement when they collide with air molecules [84]. According to the International Commission on Radiological Protection (IRCP) 1994 model for particle deposition in the respiratory tract [85], inhaled particles deposit in various segments of the human respiratory tract that can be grossly divided into three anatomical regions: the nasopharyngeal, tracheobronchial and alveolar regions [85]. Once particles get deposited in the lungs, they could trigger or enhance ROS formation when in contact with epithelial cells, alveolar macrophages, pneumocytes, etc. as discussed in the previous section. This could lead to development of interstitial and/or alveolar inflammation with the subsequent release of inflammatory mediators into the systemic circulation. While particle deposition (step a) has been amply studied in both animals and humans as well as their ability to induce free radical reactions in pulmonary cells (step b), the remaining steps c and d are still awaiting definite confirmation.

There is clear evidence that particle deposition can lead to systemic inflammatory events. Suwa et al reported that their PM_10_-exposed animals exhibited elevation of polymorphonuclear cells and circulating band cell counts 2 weeks after starting the exposures, with an increase in the size of the bone marrow mitotic pool as assessed by BrdU-labeling [38]. Although they claimed that their work support the notion of PM-induced systemic inflammation in response to local pulmonary inflammation, they did not provide evidence of increased systemic proinflammatory markers that could be linked to both effects. Likewise, none of the CAPs studies provided direct evidence for such mediators in apoE^-/- ^mice. However, a recent study of C57BL6 mice exposed to CAPs for 6 months did result in increased plasma concentrations of inflammatory biomarkers such as tumor necrosis factor-α (TNF-α), interleukin-6 (IL-6), E-selectin and intracellular adhesion molecule-1 (ICAM-1), indicative of systemic inflammatory effects [86]. In that study, PM_2.5 _exposures also led to increased visceral adiposity and insulin resistance, suggesting a possible link between air pollutants and type 2 diabetes mellitus [86]. Despite the lack for direct evidence of systemic inflammatory mediators in apoE^-/- ^mice, Araujo et al showed that CAPs exposures led to the development of dysfunctional HDL with a decrease or loss of its anti-inflammatory properties as exhibited by PM_2.5 _and UFP-exposed mice, respectively [44]. Furthermore, UFP exposures led to HDL that exhibited proinflammatory properties and could therefore contribute to enhanced disease pathogenesis (Figure 6).

PM-mediated generation of dysfunctional HDL could boost and propagate systemic inflammatory effects or at least function as a marker of systemic inflammation. It is well established that plasma HDL cholesterol and apoA1 levels are inversely correlated with the risk for coronary artery disease [87-89]. In addition to the well-characterized ability to promote reverse cholesterol transport [90], HDL has been reported to have antioxidant, antiinflammatory and antithrombotic activities [91-93]. However, high levels are not always protective in subjects, suggesting that not all HDLs prevent atherosclerosis [94]. Furthermore, dysfunctional proinflammatory HDL may serve as a useful marker for predicting susceptibility for atherosclerosis in humans [95] and in rabbits [96], where it has been found to be a better predictor han total or LDL cholesterol levels. Thus, it will be necessary to characterize the type of alterations that PM exposures induce in HDL particles and confirm whether those happen in human subjects also. This can help to elucidate pathogenesis and may even lead to the identification of a potential biomarker.

**Figure 7:**
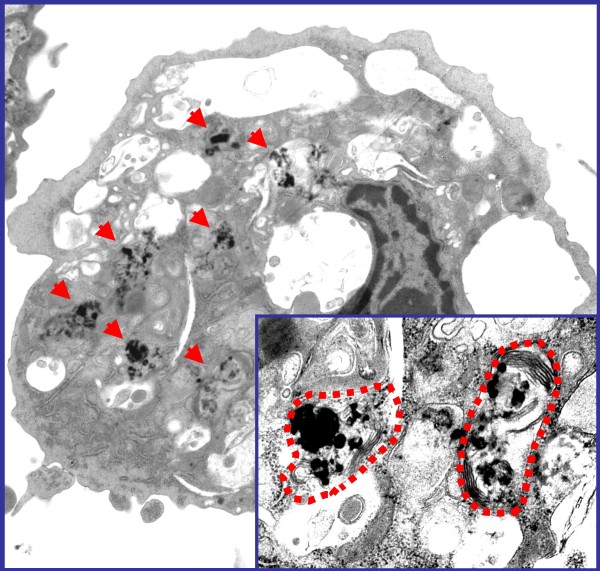
**UFP are endocytosed by alveolar macrophages**. Electron microscopy photographs of a lipid-laden alveolar macrophage harvested from an apoE^-/- ^mouse exposed to concentrated ambient ultrafine particles (<0.18 μm). Red arrows indicate electron-dense material corresponding to intracellular aggregates of nanoparticles. Notice the subcellular mitochondrial localization encircled by the red dotting. Source: Journal cover of Araujo et al [44].

Interestingly, these proinflammatory effects occurred in the absence of significant pulmonary inflammation which raises the possibility that the lungs might not need to be an active player or mediator in the induction of systemic vascular inflammation. Alternatively, particles could be endocytosed by alveolar macrophages (Figure 7), which could trigger activation of molecular inflammatory pathways that could be transduced systemically even in the absence of obvious alveolitis or interstitial pneumonitis. Another potential mechanism is that once particles get deposited in the lungs, they could undergo transcytosis across epithelia of the respiratory tract into the interstitium and access the blood circulation directly or via lymphatics (Figure 6), or could be taken up by sensory nerve endings embedded in airway epithelia, followed by axonal translocation to ganglionic and CNS structures [84]. While there are multiple reports that support the notion of systemic translocation of synthetic nanoparticles in experimental models [52,97,98] and humans [99], no convincing demonstration has been provided about this route for inhaled ambient particles. In addition, in case nanoparticles do translocate to the systemic circulation, no studies have shown that the translocated particles actually reach or deposit in blood vessels, perhaps due to the inability to detect them at these sites because of small size. There could be alternative ports of entry such as ingestion and absorption via the gastrointestinal tract or direct contact through dermal exposure [84]. However, there is no direct evidence for the significance of these routes in the exposure to ambient particles and although their feasibility has been supported by studies with engineered nanoparticles, these are particles that fall outside the scope of this review.

An alternative possibility is that particles deposited in the lungs get internalized into the cells (e.g. endocytosis by alveolar macrophages) and their chemicals could get translocated into the systemic circulation allowing them to reach target tissues (Figure 6). Whether the whole particles or chemical constituents access the systemic targets, they can activate important proatherogenic molecular pathways once in contact with cells from the vascular endothelium [60]. Although we have not found direct evidence for increased aortic oxidative stress, we showed that both PM_2.5 _and UFP exposures led to increased hepatic oxidative stress [44]. In addition, UFP triggered the upregulation of Nrf2-regulated antioxidant genes and unfolded protein response (UPR) genes in the liver, suggesting that PM exposure leads to both systemic prooxidant and proinflammatory effects [44].

It is clear that inhaled particles do get deposited in the lungs (step a), with the potential to exert prooxidative effects and activate molecular pathways in response (step b), even in the absence of obvious clinical inflammation (step c). Somehow, systemic vascular inflammation and prooxidative effects follow via a mechanism(s) still to be elucidated. In addition, oxidative stress and inflammation are strongly connected and either one can lead to the other. It is likely that the prooxidant potential of ambient particles determine their ability to activate pathways that lead to prooxidant and proinflammatory effects in the vasculature and promotion of atherosclerosis. However, in spite of this logical inference, it still remains unclear whether PM-induced prooxidant effects trigger or are the consequence of vascular inflammatory effects. Further research is required to elucidate these mechanisms and to prove whether particle-mediated proinflammatory effects are due indeed to its prooxidant potential.

## Why are UFP more proatherogenic than PM2.5?

**Figure 8:**
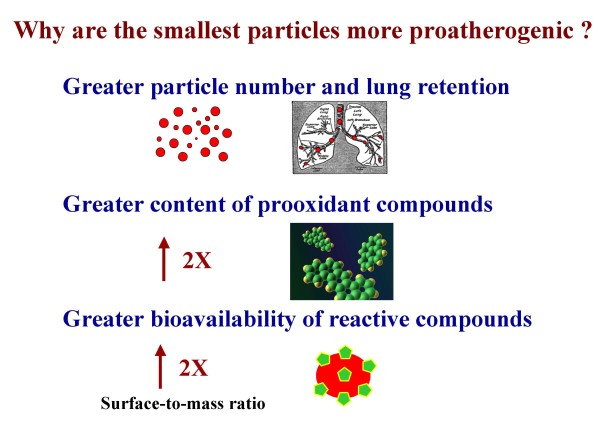
**Factors that may explain greater UFP's proatherogenic potential**. UFP (in red) are the smallest, most numerous particles and with best pulmonary retention (top panel). UFP exhibit greater relative content of redox-active compounds (e.g. PAHs, in green) than bigger particles (mid panel). UFP's greater surface-to-mass ratio may allow reactive compounds (in green) to have increased localization towards the surface of the particle (in red) and be more bioavailable for free-radical reactions when in contact with cells (bottom panel). Estimates for the increased content of prooxidant PAHs and surface-to-mass ratio in comparison to PM_2.5_are from the SCPC study [44].

A number of factors other than particle size need to be considered to explain why UFP may be more atherogenic than PM 2.5 (Table 4, Figure 8). This includes consideration of particle numbers, physicochemical composition, prooxidant potential, bioavailability and pulmonary retention. A brief discussion of how these factors could contribute to the increased atherogenic potential of UFP include:

### a) Larger particle number

Exposure to PM has been traditionally assessed by measures of mass per space (e.g. daily, annual, average or peak μg/m^3^). Adverse health effects have been correlated linearly with various measures of exposure, without any clear threshold below which PM levels have been found to be consistently safe [7,8,100]. However, particles < 0.1-0.2 μm, which contribute very little to overall PM_2.5 _mass, represent > 85-90% of the total PM_2.5 _particle number [101]. Therefore, it is conceivable that larger particle numbers in UFP atmospheres, despite a smaller mass, could result in larger biological effects. In our study, development of larger atherosclerotic lesions in the UFP exposures correlated with increased particle numbers rather than with PM mass. Thus, the 25% increase in atherosclerotic lesion area in UFP vs. PM_2.5_-exposed mice occurred in parallel with a 44% increase in the number of particles < 0.18 μm in spite of a 4-fold smaller PM mass [44].

### b) Larger content of redox active compounds

PM is composed of hundreds of organic chemicals and transition metals that could trigger or exacerbate free radical reactions contributing to systemic proatherogenic effects. As discussed in section 3, UFP are enriched in organic carbon content as well as prooxidative PAHs (Table 4) that promote oxidative stress and inflammation. This could explain the increase in atherosclerotic lesion development. Whether or not these compounds are the actual toxicants contributing to atherogenesis, they may serve as a proxy for the substances ultimately involved. For instance, PAHs can be converted to quinones and other redox cycling substances. In addition, it appears that while transition metals are also important catalysts for free radical reactions *in-vitro*, they may be less critical for the *in-vivo *systemic effects, as UFP exhibited greater proatherogenic potential despite a lower metal content than PM_2.5 _[44].

### c) Greater bioavailability

An important feature derived from the small size of UFP is that their large surface-to-mass ratio along with increased number could lead to a sizable increase in bioavailable surface [102]. The number of atoms or molecules that are displayed and packaged on the surface of particles increase exponentially as the size shrinks below 100 nm [84]. Thus, the presence of bioreactive chemicals (e.g. PAHs, transition metals) on the large surface area of UFP could make these chemicals more bioavailable at the contact sites of the particles with cells and tissues where prooxidant effects take place (Figure 8). The surface area or more accurately the bioreactive surface area of UFP constitutes an exposure parameter that may more accurately depict small particle dosimetry than the current calculation of PM exposure by mass [31,103,104]. Unfortunately there are no easy ways to calculate reactive surface area at present.

### d) Greater lung retention

Small particle size allows better penetration and diffusion into the lungs according to the IRCP 1994 model for particle deposition in the respiratory tract [85]. The fractional deposition of particles during nose breathing into the three anatomical regions in this model (nasopharyngeal, tracheobronchial and alveolar regions) varies significantly depending on the aerodynamic diameter of the inhaled particles, respiratory frequency, pattern of breathing and respiratory co-morbid conditions. Indeed, UFP exhibit a greater fractional deposition in the tracheobronchial and alveolar regions than larger particles. For instance, 20-nm synthetic particles, which correspond to the particle peak size of ambient urban aerosols, exhibit the highest fractional deposition efficiency in the alveolar region (~50%) [84,102]. In addition, the UFP fractional deposition increases markedly during exercise in healthy subjects [105] when the respiratory frequency is greater and the pattern of breathing is altered. Furthermore, pulmonary conditions such as chronic obstructive pulmonary disease (COPD) or asthma that are characterized by airway constriction with changes in air flow dynamics and increases in lung residual volume, have been shown to result in increased fractional deposition of UFP [106,107], likely as a result of greater contact with the airway wall and enhanced diffusional deposition. Better airway deposition could translate into better retention, cellular uptake and greater propensity to induce systemic effects, a notion that remains to be proven. The greater retention of UFP could be partly due to increased Van der Waals forces or electrostatic interactions, all of which contributes to "adhesive interactions" [108,109]. Once deposited in the alveoli, particles are wetted by the surfactant film and could be taken up by alveolar macrophages by physico-chemical forces rather than receptor-ligand interactions that play a role in phagocytosis of larger particles. Whether these particles or chemical constituents enter the systemic circulation is a matter of intense debate that requires further investigation. It will be important to determine whether all these factors confer greater toxicity to UFP in human subjects since they may imply the need for adjusting the metrics of exposure to take account of particle number, surface area and oxidant potential.

## Relationship of PM to other proatherogenic factors: Gene-environment interactions

The development of PM-induced proatherogenic effects likely depends not only on exposure characteristics such as dose, duration and location of exposure as discussed above but also on the differential susceptibility of exposed individuals [110], according to a gene-environmental paradigm that recognizes that atherosclerosis is a complex disease that involves a multiplicity of genetic and environmental factors [73]. In fact, exposure to PM is now considered as a novel risk factor that could contribute to the composite clinical risk in synergy with well known cardiovascular risk factors such as hypertension, hypercholesterolemia, smoking, diabetes and/or obesity among others. However, this synergy has only been studied to a limited degree. While subjects with advanced age [111,112], obesity [19] or congestive heart failure [113] have been reported to exhibit increased susceptibility to PM-mediated cardiovascular effects, only one study among those evaluating the impact of PM on atherosclerosis found predisposing conditions such as age and sex [33]. However, the number of studies and number of subjects are very small and it is still too early to draw any conclusions. Further research is required with larger number of individuals that undergo extensive characterization of clinical traits to maximize the possibility of unveiling gene-environment interactions.

Cellular and experimental animal work can be instrumental to identify conditions that help establish the molecular and genetic pathways that are involved in the interaction of PM with other risk factors. While the individual contribution of air pollutants to atherogenesis might be small, their effects could be exacerbated in synergy with other known proatherogenic factors. These interactions could be more evident on molecular pathways known to mediate disease pathogenesis. One possibility is that PM proatherogenic effects depend on generation of oxidative stress. PM could exacerbate the biological activity of well-known prooxidative and proatherogenic factors such as ox-LDL. We tested this hypothesis by evaluating the antioxidant response of microvascular endothelial cells to DEP in the absence and presence of oxidized PAPC (1-palmitoyl-2-arachidonyl-sn-glycero-3-phosphorylcholine), one of the key prooxidative components generated in LDL particles. While both DEP and ox-PAPC have been shown to exert prooxidative effects in vascular cells [58,59], the combination of both stimuli synergize in increasing antioxidant gene expression, including HO-1 [60]. In addition, analysis of the genomic profiles of cells subjected to the various treatments unveiled a large number of genes where the same DEP and ox-PAPC synergy was present. These genes were grouped into clusters that were enriched in proinflammatory, apoptotic and UPR pathways, all important players in atherogenesis. These gene clusters are reminiscent of the different elements of the hierarchical oxidative stress paradigm discussed earlier on. Some representative examples include proinflammatory genes such as Interleukin 8 (IL-8) and chemokine (C-X-C motif) ligand 1 (CXCL1) production, immune response genes such as Interleukin 11 (IL-11) and UPR genes such as activating transcription factor 4 (ATF 4), heat shock 70 kDa protein 8 (HSPA8) and X-box binding protein 1 (XBP1). Importantly, some of these same genes were found to be preferentially upregulated in the livers of apoE^-/-^mice exposed to UFP, supporting the validity of *in-vitro *generated data to predict relevant *in-vivo *outcomes.

The identification of participant gene pathways would pave the way to study gene-environment interactions in the development of PM proatherogenic effects. For instance, it is conceivable that modulation in the expression levels of antioxidant genes as a result of gene polymorphisms could alter the magnitude of effects caused by PM. Indeed, polymorphic variants of the glutathione-S-transferase genes GSTP1 and GSTM1 [114] or HO-1 [115] have been reported to influence the risk of developing new onset asthma associated to air pollutants. It appears that gene-gene and gene-environment interactions could determine the fate of individuals to develop new disease under specific circumstances. Thus, in a population-based cohort study in Southern California, child carriers of the short GT-repeat polymorphism that results in higher HO-1 expression, exhibited decreased risk of new-onset asthma as compared with carriers of the non short GT alleles [115]. This association was only present in non-hispanic white children but not in hispanic subjects and especially among those residing in low ozone communities. These findings illustrate the high level of complexity present in the interaction between genetic and environmental factors that could be similar to the interactions between particulate pollutants and atherosclerosis-related genes. Interestingly, one study reported inverse associations of exposure to primary aerosols with the antioxidant erythrocyte enzyme Cu/Zn-SOD and GPx-1 in subjects with coronary artery disease [36]. Polymorphisms and variations in the expression of these phase 2 enzymes could potentially lead to increased susceptibility to adverse cardiovascular events. It is therefore necessary to perform large-scale studies where in addition to detailed characterization of pollutant exposures, individuals are genotyped and extensively phenotyped for clinical traits and gene expression levels. This will enhance our understanding of individual susceptibility to PM-mediated adverse cardiovascular effects and may also shed light on the adaptation mechanisms of people who live in a polluted environment.

## Multi-pollutant effects contributing to atherosclerosis

Although most of the published data attributes the cardiovascular effects to the particulate matter components, these associations have been questioned on the basis of publication bias. While it is extremely unlikely that publication bias could explain the overall consistency, coherence and strength of those associations [6,116], it is conceivable that other co-pollutants could play a role. Work from the Lovelace Respiratory Institute has shown that exposure of apoE^-/- ^mice to both whole and particle-free gasoline exhausts results in the induction of aortic metalloproteinases (MMP)-2 and -9 after 7 weeks [117] and as early as after 7 days [118]. This suggests that gaseous components are responsible for those effects. Interestingly, gasoline exhaust emissions appear to trigger both ROS and endothelin 1 (ET-1) mediated pathways with a complex degree of interconnectivity. Increased expression and activity of MMP-9 was shown to be ET-1 mediated and also present in a small cohort of humans exposed to diesel exhausts [118]. While ET-1-mediated pathways triggered by either gasoline and/or diesel exhausts could play a role in atherogenesis, increased MMP-9 could be modulating plaque stability [119] and induce plaque rupture and subsequent development of acute coronary syndromes [120]. It is possible thus that gaseous and particulate pollutants could exert different but cooperative effects, which will need to be explored in more details in the future.

## Future research perspectives

We have seen in recent years an enormous improvement in our understanding of the cardiovascular effects of air pollution. Human epidemiological studies together with experimental animal data discussed above support a causal association between air PM and atherosclerosis. Consequently, PM exposure is now considered a novel risk factor [5]. Much work is still needed however, in the identification of those factors that are clinically relevant. This work will require the collaboration of investigators from diverse disciplines that span from epidemiology, toxicology and engineering to pathology, genetics, vascular and molecular biology. Better understanding of the toxic air components, toxicology and pathogenesis will allow us to formulate better ways to monitor and control air pollution as well as to avoid or decrease their effects on cardiovascular health.

Although most of the epidemiological work has been focused on PM mass exposure parameters of the various size fractions, other physicochemical parameters such as chemical composition, particle number, surface area, surface-to-mass area need to be considered in future studies as discussed above. It may require us to develop new parameters or composite metrics that can combine all these various factors to best capture the overall PM-related cardiovascular toxicity. This may help to elucidate why exposure to PM, in strong association with CV events in the Northeast, Midwest and Southern U.S. regions, appears not be associated with excess risk in the Northwestern U.S. regions [121,122].

Animal data that supports the notion of UFP's greater proatherogenic potential and development of HDL dysfunction are based on one single study [44] and require confirmation by further studies, including in human subjects. Dose-response studies are also needed to determine various toxicological parameters (e.g. threshold, latency of effects, etc.) and whether proatherogenic effects are in relation to the whole burden of PM exposure or the kinetics of those exposures. Since air pollutants include hundreds of different compounds, it is unlikely that one single or a few chemical constituents could be responsible for all pathogenic effects but it could be possible to identify groups of compounds that work in an additive or synergistic fashion (e.g. PAHs).

While exposure to PM leads to both prooxidant and proinflammatory effects, there is a great degree of interconnection in between oxidative stress and vascular inflammation that makes it very difficult to determine whether the induction of vascular oxidative stress precedes or follows inflammation as discussed above. The use of antioxidants as well as transgenics or knockouts for critical antioxidant genes or transcription factors (e.g. Nrf2, HO-1, superoxide dismutase) or prooxidant genes (e.g. NADPH oxidase) may help to clarify this. Since the portal of entry may determine the pathogenic mechanism and the mode for transduction into systemic effects, it will be important to fully characterized the role of the lungs as a mediator and to assess alternative ports of entry such as the gastrointestinal tract by ingestion of particles or skin by contact.

Exposure to PM likely leads to important gene-environment interactions that will need to be addressed in both human and animal studies to better define conditions of increased susceptibility. The use of gene profiling, analysis of gene expression by system biology approaches, genetically-engineered animals as well as mouse and human whole genome association approaches are needed to identify genes and/or pathways that either mediate the effects of air pollutants, are influenced by them or could be targeted for intervention in the future.

## Conclusions

Cumulative epidemiological data support the association of exposure to air pollution with cardiovascular morbidity and mortality, mostly in relation to its particulate matter components. Experimental animal work using hypercholesterolemic rabbits and apoE null mice shows that ambient PM exposure promotes atherosclerosis and that the smaller the particles, the greater the proatherogenic effects. Enhancement of atherosclerosis correlates with the induction of systemic prooxidant and proinflammatory effects although the mechanism for transduction of these effects is not clear. UFP particles may be more toxic based on their greater number, larger content of redox active compounds such as PAHs, greater surface-to-mass ratio and bioavailability of chemically active constituents. Much work is needed to better characterize the main toxic compounds, mechanism(s) of pathogenesis, types of genetic susceptibility that exposed individuals may exhibit, relevant gene-environment interactions in the induction of those effects and development of a biomarker of exposure, degree of damage or susceptibility.

## Competing interests

The authors declare that they have no competing interests.

## Authors' contributions

Both JAA and AEN participated in the conception, design, drafting and critical revision of the manuscript. Both authors read and approved the final manuscript.
